# Integrating CAR-T therapy with PD-1/PD-L1 blockade: Mechanisms, synergy, and optimized strategies in NSCLC

**DOI:** 10.1016/j.isci.2025.114607

**Published:** 2026-01-14

**Authors:** Xingxing Li, Zitong Wang, Shuyang Mao, Yijun Zhao, Jiayi Ye, Beibei Liang, Jialei Peng, Xinyi Xie, Jinglan Pan, Chunhai Xiao, Jian Zhao, Xiuhong Lu, Wei Xie

**Affiliations:** 1 Graduate School, Shanghai University of Traditional Chinese Medicine, Shanghai, China; 2 Digital and Intelligent Empowerment Biomedical Innovation Center, School of Pharmacy, Shanghai University of Medicine and Health Sciences, Shanghai, China; 3 Shanghai Key Laboratory of Molecular Imaging, Shanghai University of Medicine and Health Sciences, Shanghai, China; 4 School of Health Science and Engineering, University of Shanghai for Science and Technology, Shanghai, China; 5 Shanghai University of Traditional Chinese Medicine, Shanghai, China; 6 Jinshan Branch of Shanghai Sixth People’s Hospital, Shanghai, China

**Keywords:** Health sciences

## Abstract

Non-small cell lung cancer (NSCLC) presents persistent challenges in immunotherapy, as the clinical benefit of programmed cell death protein 1 (PD-1) and programmed death-ligand 1 (PD-L1) inhibitors is frequently constrained by intrinsic and acquired resistance. Central contributors include impaired antigen presentation, T cell exclusion, and the accumulation of immunosuppressive populations that collectively establish a “cold” tumor microenvironment (TME). These mechanisms dampen cytotoxic CD8^+^ T cell function and limit the durability of PD-1/PD-L1 blockade. Our single-cell RNA-seq reanalysis further supports that exhausted CD8^+^ T cells and regulatory T cells (Tregs) are enriched in non-responsive NSCLC, accompanied by compensatory upregulation of alternative checkpoints.

Given these limitations, complementary approaches such as chimeric antigen receptor T cell (CAR-T) therapy have shown promising potential to overcome PD-1/PD-L1-driven immunosuppression. Although CAR-T cells are effective in hematologic malignancies, their activity in NSCLC is limited by antigen heterogeneity, dysfunction induced by the TME, and inhibitory signaling mediated by PD-1. Integrating checkpoint blockade with CAR-T therapy offers a rational strategy: PD-1/PD-L1 inhibitors can alleviate exhaustion and remodel the TME, and CAR-T cells provide potent, antigen-specific cytotoxicity and enhance infiltration into poorly immunogenic tumors.

This review summarizes mechanistic intersections between PD-1/PD-L1 signaling and CAR-T cell biology and discusses emerging synergistic strategies, including multi-target CAR constructs, engineering strategies targeting the TME and tumor metabolism, and localized or self-delivered checkpoint blockade. We also highlight safety-oriented designs, including logic-gated CARs and inducible safety switches, which aim to mitigate cytokine-related or on-target/off-tumor toxicities. Finally, we outline how computational modeling and machine learning may accelerate the design, optimization, and personalized application of these combination approaches. Together, the integration of CAR-T therapy with PD-1/PD-L1 inhibition represents a promising framework for overcoming resistance and improving outcomes in NSCLC.

## Graphical abstract

**Figure:**
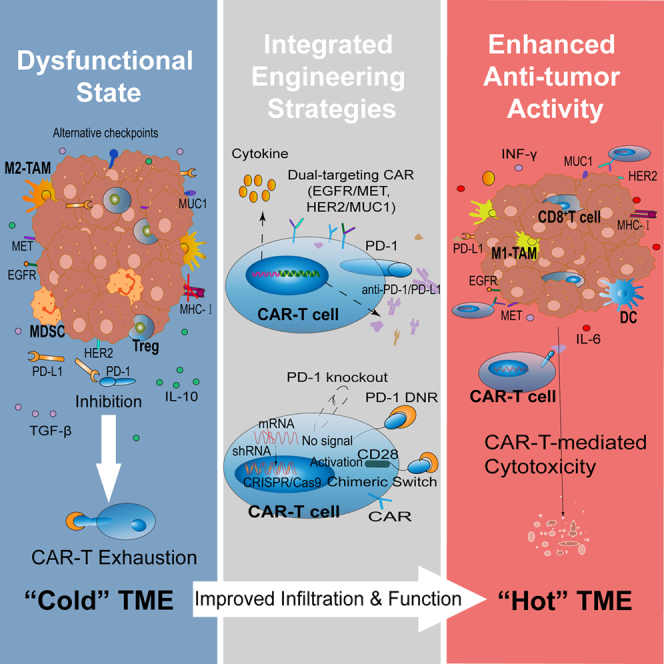

## Introduction

The interaction between programmed cell death protein 1 (PD-1) and programmed cell death ligand 1 (PD-L1) is crucial for how cancer cells evade immune surveillance. Their binding deactivates T cells, allowing tumors to avoid immune-mediated elimination.^1^ PD-1/PD-L1 inhibitors work by blocking this connection, thereby enabling T cells to resume their anti-tumor activity. However, resistance to these inhibitors develops in some patients with non-small cell lung cancer (NSCLC). This resistance can arise from a weak immune response within the tumor, reduced surface MHC expression, and dysfunctional interferon-gamma (IFN-γ) signaling, all of which contribute to a “cold” tumor microenvironment (TME) characterized by limited T cell presence.^2^^,^^3^ Therefore, modulating the immune environment or enhancing T cell recruitment could improve the efficacy of PD-1/PD-L1 treatments.

Chimeric antigen receptor T cell (CAR-T) therapy is an advanced immunotherapy that involves engineering a patient’s T cells to target specific cancer antigens and reinfusing them to attack the disease. These modified CAR-T cells are expanded outside the body and can function independently of MHC recognition, mitigating the impact of MHC downregulation by cancer cells.^3^^,^^4^ This approach has achieved significant success in treating blood cancers.^5^ For solid tumors, however, CAR-T therapy encounters challenges including antigen heterogeneity, physical barriers, and the immunosuppressive TME, which lead to T cell exhaustion and limited efficacy.^6^

PD-1/PD-L1 inhibitors and CAR-T cell therapy each present distinct advantages and limitations in the treatment of NSCLC, and their efficacy is closely influenced by the tumor microenvironment. “Hot” tumors generally respond better to PD-1/PD-L1 inhibitors, whereas “cold” tumors remain more difficult to treat with these agents.^7^ CAR-T cells can enhance immune infiltration within tumors, particularly in “cold” lesions with low immunogenicity, and can partially disrupt the prevailing immunosuppressive microenvironment; complementarily, PD-1/PD-L1 inhibitors help relieve CAR-T cell exhaustion and modify immunosuppressive conditions in a way that supports more durable anti-tumor activity.^8^ Therefore, combining CAR-T therapy with PD-1/PD-L1 pathway inhibition may provide complementary benefits in NSCLC, especially for patients exhibiting high PD-L1 expression.

## Challenges of PD-1/PD-L1 inhibitors in NSCLC

Immunotherapy directed at the PD-1/PD-L1 axis has reshaped the treatment landscape of NSCLC and is now a standard option for advanced disease. However, its broader clinical utility is limited by primary resistance, acquired resistance, and immune-related adverse events (Table 1). In unselected NSCLC populations, anti-PD-1/PD-L1 monotherapy yields objective response rates below 20%, and outcomes are even poorer in patients with epidermal growth factor receptor (EGFR) or anaplastic lymphoma kinase (ALK) alterations. Many patients who initially benefit eventually relapse because of acquired resistance.^29^ PD-L1 expression in tumor or immune cells remains an important yet imperfect predictor of therapeutic efficacy.^30^ Increasing evidence indicates that multiple components of the TME regulate responses to PD-1/PD-L1 blockade, and impaired interactions between tumor and immune cells frequently underlie treatment resistance.^31^

**Table 1:** Core mechanisms of PD-1/PD-L1 inhibitor resistance in NSCLC

Category	Study	Mechanistic Pathway	Functional Effect
Antigen Presentation Defects	Ricciuti et al.^9^; Restifo et al.^10^	Downregulation of MHC-Ⅰ expression or disruption of peptide loading via *B2M* deletion.	Reduced antigen presentation and tumor immunogenicity; impaired T cell recognition
	Horn et al.^11^	Impaired IFN-γ signaling (due to mutations in JAK1/2/STAT1/2/IRF1) suppressing MHC-Ⅰ-mediated antigen presentation and PD-L1 expression.	
	Yang et al.^12^	Elevated itaconate (induced by PD-1 inhibitor-triggered IFN-γ) impairing dendritic cell function.	
Effector T Cell Exclusion	Exposito et al.^13^	PTEN loss activating PI3K/AKT/mTOR pathway, promoting TGF-β/CXCL10 secretion to inhibit CD8^+^ T cells and expand Tregs.	Poor CD8^+^ T cell infiltration; “cold” TME; resistance to PD-1 blockade
	Muto et al.^14^	Wnt/β-catenin pathway suppressing CCL4 production, reducing CD103^+^ dendritic cell recruitment and T cell infiltration.	
	Mariathasan et al.^15^; Li et al.^16^; Du et al.^17^	TGF-β1-induced LN-γ2 forming physical barriers; TGF-β upregulating PD-L1 via MRTF-A-NF-κB/p65 axis.	
Infiltration of Immunosuppressive Cells	Liu et al.^18^	Tregs secreting TGF-β/IL-10/IL-35/adenosine and expressing PD-L1 to inhibit effector cells.	Suppression of effector T and NK cell activity; reinforcement of immunosuppressive network
	Zhang et al.^19^; Liang et al.^20^; Zhao et al.^21^	MDSCs depleting amino acids, producing NO/ROS, and promoting Treg expansion via TGF-β/IL-10.	
	Wang et al.^22^; Pu et al.^23^; Lv et al.^24^	M2-TAMs secreting TGF-β/IL-10, upregulating PD-L1, and intercepting anti-PD-1 mAbs via Fcγ receptors.	
Regulation by Alternative Immune Checkpoints	–	Compensatory upregulation post-ICI treatment, forming alternative inhibitory pathways to induce T cell exhaustion.	T cell exhaustion and adaptive resistance to PD-1/PD-L1 inhibitors
	Cheng et al.^25^; Skoulidis et al.^26^	CTLA-4 inhibiting DC co-stimulation.	
	Kalinka et al.^27^	LAG-3 binding MHC-Ⅱ.	
	Zhang et al.^28^	TIM-3/TIGIT associating with T cell dysfunction.	

MHC, Major histocompatibility complex; B2M, beta-2 microglobulin; IFN-γ, interferon-gamma; JAK1/2, janus kinase 1 and 2; STAT1/2, signal transducer and activator of transcription 1 and 2; IRF1, interferon regulatory factor 1; PD-L1, programmed death-ligand 1; PD-1, programmed cell death protein 1; PTEN, phosphatase and tensin homology deleted on chromosome ten; PI3K, phosphatidylinositol 3-kinase; AKT, protein kinase B; mTOR, mechanistic target of rapamycin; TGF-β, transforming growth factor-beta; CXCL10, C-X-C motif chemokine ligand 10; CCL4, C-C motif chemokine ligand 4; LN-γ2, laminin subunit gamma-2; MRTF-A, myocardin-related transcription factor A; NF-κB, nuclear factor kappa-light-chain-enhancer of activated B cells; TME, tumor microenvironment; IL-10, interleukin-10; IL-35, interleukin-35; MDSCs, myeloid-derived suppressor cells; NO, nitric oxide; ROS, reactive oxygen species; M2-TAMs, M2-type tumor-associated macrophages; mAbs, monoclonal Antibodies; Fcγ, fragment crystallizable gamma; NK, natural killer; ICI, immune checkpoint inhibitors; CTLA-4, cytotoxic T lymphocyte-associated antigen-4; DC, dendritic cell; LAG-3, lymphocyte activation gene 3; TIM-3, T cell immunoglobulin and mucin-domain containing-3; TIGIT, T-cell immunoreceptor with Ig and ITIM domains.

The TME includes all non-malignant elements within and around the tumor mass.^32^ NSCLC tumors are often categorized as cold or hot based on immune cell density, spatial distribution, and functional activity, which helps predict responsiveness to immunotherapy.^29^ Hot tumors generally exhibit higher tumor mutational burden, elevated PD-L1 expression, and greater immune infiltration and are commonly associated with KRAS mutations or oncogene-wild-type NSCLC. These features correspond to better initial responses to PD-1/PD-L1 inhibitors, although most patients ultimately develop resistance due to mechanisms such as activation of alternative checkpoints, reduced antigen recognition, T cell exhaustion, or metabolic dysregulation.^7^^,^^33^^,^^34^ In contrast, cold tumors typically show low tumor mutational burden, reduced PD-L1 levels, and limited immune infiltration.^35^ These characteristics are frequently present in EGFR-mutant or ALK-rearranged NSCLC and contribute to intrinsic resistance to checkpoint blockade.^33^^,^^34^ Key drivers of resistance in cold tumors include insufficient infiltration of effector immune cells, weak tumor immunogenicity, and defects in antigen presentation.^29^

### Defects in antigen presentation

Major histocompatibility complex (MHC) class I molecules play a central role in presenting tumor antigens to CD8^+^ T cells. When MHC-I expression is absent or reduced, antigen visibility declines, enabling tumor cells to evade immune surveillance.^36^^,^^37^ Tumor cells may suppress MHC-I surface expression or impair peptide loading through alterations such as beta-2 microglobulin *(B2M)* gene deletion.^9^^,^^10^ In a longitudinal study of 82 NSCLC patients treated with immune checkpoint inhibitors (ICIs), 27.8% developed acquired resistance-associated mutations, including loss-of-function alterations in *B2M*. These mutations emerged only after ICI therapy and were not detected in patients receiving chemotherapy or targeted therapy, confirming *B2M* dysfunction as an ICI-specific mechanism of immune escape.^9^

Following antigen recognition via MHC-I, CD8^+^ T cells release IFN-γ, which activates the janus kinase 1 and 2 (JAK1/2)-signal transducer and activator of transcription 1 and 2 (STAT1/2) signaling cascade in tumor cells. This pathway enhances MHC-I expression and thereby supports antitumor immunity; however, it simultaneously induces PD-L1 expression, creating a counter-regulatory mechanism that enables NSCLC cells to resist immune attack.^38^ Loss-of-function mutations in JAK1/2 or STAT1/2 disrupt IFN-γ signaling, leading to insufficient PD-L1 induction and impaired MHC-I-mediated antigen presentation, both of which diminish responsiveness to PD-1/PD-L1 inhibitors.^11^ CRISPR-based studies further demonstrate that interferon regulatory factor 1 (IRF1), a key transcription factor downstream of IFN-γ, is indispensable for maintaining tumor immunogenicity. Loss of IRF1 compromises PD-L1 expression and antigen presentation, limiting T cell activation and infiltration and ultimately reducing immunotherapy efficacy.^39^^,^^40^ Additionally, PD-1 blockade has been shown to stimulate CD8^+^ T cells to secrete IFN-γ, which activates the JAK-STAT1 pathway in macrophages and increases itaconate synthase expression. Elevated itaconate suppresses dendritic cell antigen presentation, indirectly weakening CD8^+^ T cell responses and contributing to anti-PD-1 resistance.^12^

### Effector T cell exclusion

Oncogenic signaling pathways within tumor cells frequently contribute to immune evasion by limiting effector T cell infiltration into the TME.^41^ Loss of phosphatase and tensin homology deleted on chromosome ten (PTEN) expression is associated with increased T cell infiltration in certain tumor regions, yet patients whose tumors lack PTEN often experience poorer clinical outcomes.^13^^,^^42^ In NSCLC, PTEN deficiency activates the phosphatidylinositol 3-kinase (PI3K)/protein kinase B (AKT)/mechanistic target of rapamycin (mTOR) signaling pathway. This activation drives the secretion of transforming growth factor β (TGF-β) and C-X-C motif chemokine ligand 10 (CXCL10), which promotes the accumulation of regulatory T cells (Tregs) while suppressing CD8^+^ T cell activity. This shift results in a fibrotic, highly metastatic TME that diminishes the effectiveness of anti-PD-1 therapy. Supporting evidence indicates that patients with these molecular features typically exhibit elevated PD-L1 and PD-L2 expression and worse progression-free survival.^13^ PTEN-deficient tumors also commonly harbor serine/threonine kinase 11 *(STK11)*, *B2M*, or *JAK1/2* mutations, which further impair antitumor immunity and contribute to a “cold” tumor phenotype characterized by high PD-L1 levels and exclusion of T cells, ultimately promoting resistance to PD-1/PD-L1 blockade.^9^^,^^13^

T cell exclusion can also arise from aberrant activation of the Wnt/β-catenin pathway.^43^ In one clinical case, a patient who initially responded to PD-1/PD-L1 inhibitors later developed metastatic progression accompanied by elevated β-catenin expression and a marked reduction in T cell infiltration.^44^ Mechanistically, Wnt/β-catenin activation induces activating transcription factor 3 (ATF3), which suppresses C-C motif chemokine ligand 4 (CCL4) production, leading to insufficient recruitment of BATF3 (basic leucine zipper transcription factor ATF-like 3)-dependent CD103^+^ dendritic cells. This reduction limits the secretion of CXCL9, another key chemokine of the CXCL family, and CXCL10, thereby restricting effector T cell entry into tumor lesions and creating a T cell exclusion state that undermines the efficacy of PD-1/PD-L1 inhibitors.^14^

In NSCLC, TGF-β1 promotes the expression of laminin subunit gamma-2 (LN-γ2) by activating the JNK/AP-1 (c-Jun N-terminal kinase/activator protein-1) signaling pathway. The resulting LN-γ2 protein forms a protective physical barrier that obstructs T cell infiltration into the tumor nest, ultimately substantially weakening the immune response to anti-PD-1 therapy.^15^^,^^16^ In addition, TGF-β can activate non-canonical signaling pathways, such as PI3K/AKT, RhoA (Ras homolog family member A), and Wnt/β-catenin. It can also induce the nuclear translocation of the transcriptional coactivator myocardin-related transcription factor A (MRTF-A). Subsequently, MRTF-A forms a complex with the p65 subunit of the nuclear factor kappa-light-chain-enhancer of activated B cells (NF-κB) complex. This complex directly binds to the PD-L1 promoter to upregulate its transcriptional expression, thereby enhancing tumor cell immune escape.^17^

### Infiltration of immunosuppressive cells

**Figure 1:**
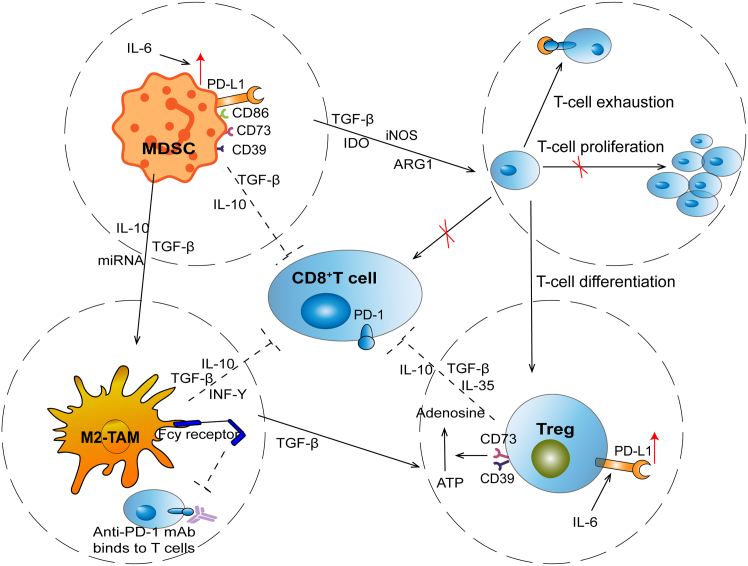
Crosstalk among immunosuppressive cells in the TME drives resistance to PD-1/PD-L1 blockade in NSCLC The TME of NSCLC is enriched with regulatory Tregs, MDSCs, and M2-TAMs. These immunosuppressive cells secrete cytokines such as TGF-β, IL-10, and IL-6, upregulate PD-L1, and release metabolites like adenosine, collectively suppressing CD8^+^T cell activation and proliferation. Tregs contribute through PD-L1 expression and adenosine generation (via CD39/CD73), MDSCs enhance PD-L1 and deplete nutrients essential for T cells, while M2-TAMs secrete inhibitory cytokines and can intercept anti-PD-1 antibodies via Fcγ receptors. Their cooperative interactions establish a suppressive network that drives CD8^+^ T cell exhaustion and resistance to PD-1/PD-L1 blockade. Dashed arrows represent cytokine-mediated signaling; red arrows denote upregulation; red “×” marks indicate inhibition.

The TME is enriched with immunosuppressive cells, including Tregs, myeloid-derived suppressor cells (MDSCs), and tumor-associated macrophages (TAMs). These cells secrete immunosuppressive cytokines, suppress the activity of effector T cells, and contribute to the formation of an immunosuppressive network, thereby diminishing the therapeutic efficacy of PD-1/PD-L1 inhibitors (Figure 1).^1^

Tregs exert their immunosuppressive functions by releasing TGF-β, interleukin-10 (IL-10), interleukin-35 (IL-35), adenosine, and by expressing high levels of PD-L1. These mechanisms directly inhibit the function of CD8^+^ T cells, Th1 cells, and natural killer (NK) cells, induce effector T cell exhaustion, and impair the responsiveness of PD-1/PD-L1 blockade.^18^ In the clinical setting, a significant presence of Tregs in the TME is strongly linked to CD8^+^ T cell exhaustion and adverse prognosis. This is particularly evident in NSCLC patients with brain metastases, where both CD8^+^ T cells and Tregs are scarce, accompanied by impaired antigen presentation and reduced lymphocyte extravasation, creating a profoundly immunosuppressive TME.^22^^,^^45^ Patients who don’t respond to PD-1/PD-L1 inhibitors tend to have higher levels of PD-1-positive regulatory Tregs. Interestingly, the PD-1 balance between CD8^+^ T cells and Tregs within the TME proves more reliable in predicting treatment outcomes than traditional markers like PD-L1 or tumor mutational burden (TMB). These PD-1/PD-L1 inhibitors can definitely give CD8^+^ T cells a new lease on life, but they might also inadvertently ramp up Treg suppression, which can lead to resistance.^46^

MDSCs promote PD-1/PD-L1 blockade resistance via diverse pathways and amplify immunosuppression by collaborating with Tregs. In NSCLC, tumor-intrinsic PD-L1 activates JAK2/STAT3 signaling, increasing interleukin-6 (IL-6) production, which recruits and enhances PD-L1 expression on MDSCs, enhancing their suppressive immune effects and tolerance to PD-1/PD-L1 blockers.^47^ MDSCs impair T cell expansion and activity through the depletion of vital amino acids within the TME. Additionally, they produce nitric oxide and reactive oxygen species, disrupting T cell receptor signaling and inducing T cell apoptosis.^19^^,^^20^^,^^21^ MDSC-derived cytokines, including IL-10 and TGF-β, directly suppress CD8^+^ T cell and NK cell activity, and promote Treg expansion, establishing an immunosuppressive cascade. They drive T cell differentiation into tumor-specific Tregs via TGF-β or costimulatory molecules like PD-L1 and CD86.^21^^,^^48^ Furthermore, MDSC-derived exosomes deliver TGF-β, IL-10, and microRNAs to further suppress dendritic cells (DCs) antigen presentation and promote M2 macrophage polarization.^48^ MDSCs and Treg co-express CD39 and CD73, catalyzing the conversion of ATP to adenosine, thus intensifying the immunosuppressive network. Clinical trials revealed a link between elevated CD39^+^ CD73^+^ MDSC presence in NSCLC and both advancing disease, plus insensitivity to PD-1/PD-L1 inhibitors.^46^

TAMs, particularly the M2 phenotype, play a crucial role in modifying the immunosuppressive tumor milieu and promoting resistance to PD-1/PD-L1 checkpoint blockade.^22^^,^^23^^,^^49^ By releasing cytokines like TGF-β and IL-10, which suppress immune responses, M2-polarized TAMs effectively dampen the cytotoxic function of CD8^+^ T cells. Via the TGF-β/Smad3 (mothers against decapentaplegic homolog 3) signaling pathway, they suppress granzyme B expression and reduce interleukin-2 (IL-2) production, which weakens T cell responses. TAMs also secrete IFN-γ to activate PI3K/AKT and JAK/STAT3 pathways, including upregulation of PD-L1 on both tumor cells and itself, thereby enhancing the immunosuppressive PD-1/PD-L1 axis and further reducing the efficacy of ICIs.^22^^,^^23^^,^^24^ In terms of spatial distribution, a higher concentration of M2-TAMs within the tumor’s core is closely tied to the resistance observed in NSCLC patients to PD-1/PD-L1 checkpoint inhibitors. This resistance is marked by elevated levels of M2-specific markers, like Bcl2 and CCL5. The exosomes secreted by TAMs carry TGF-β and microRNAs, which essentially transform the pro-inflammatory M1 macrophages into tumor-friendly M2 cells. This transformation not only suppresses antigen presentation but also hinders the infiltration of T cells.^23^^,^^50^ Furthermore, TAMs harness Fcγ receptors on their surface to intercept anti-PD-1 mAbs, thereby avoiding T cell interaction and triggering CD8^+^ T cell fatigue. TGF-β secreted by TAMs also promotes the accumulation of Tregs and suppresses Th1 responses, thereby establishing multiple layers of resistance barriers.^23^

### Regulation by other immune checkpoints

In NSCLC, resistance to PD-1/PD-L1 inhibitors can arise from tumor-mediated remodeling of the immunosuppressive TME via upregulation of alternative immune checkpoints.^51^ Activated T cells in the TME are crucial for tumor cell elimination, but resistance can develop through upregulation of cytotoxic T lymphocyte-associated antigen-4 (CTLA-4), lymphocyte activation gene 3 (LAG-3), T cell immunoreceptor with Ig and ITIM domains (TIGIT), and T cell immunoglobulin and mucin-domain containing-3 (TIM-3) on T cells.^25^^,^^52^ Following PD-1/PD-L1 inhibitor resistance, Tregs proliferate in the TME, increasing CTLA-4 expression and inhibiting DC co-stimulation of CD8^+^ T cells via CD80/CD86 binding.^25^^,^^26^ LAG-3 binding to MHC class II on antigen-presenting cells causes T cell exhaustion and inhibits effector T cell proliferation.^27^ TIGIT expression, linked to T cell exhaustion, is found on various lymphocyte subtypes.^28^ TIM-3 overexpression on T and NK cells in cancer patients correlates with reduced anti-PD-1/PD-L1 therapy responses.^53^

John V. Heymach’s research team discovered that NSCLC patients with mutations in the *STK11* or *KEAP1* (*Kelch*-like *ECH*-associated protein 1) genes showed better treatment outcomes when given a dual blockade of PD-L1 and CTLA-4 inhibitors, as opposed to relying on PD-L1 inhibitors alone. This dual immunotherapeutic approach was shown to activate CD4^+^ T cells, reprogram tumor-associated myeloid cells, modulate the TME, and enhance anti-tumor immunity, thereby mitigating PD-1/PD-L1 inhibitor resistance in these genetically defined NSCLC patients.^26^ Preclinical research indicates that TIM-3 inhibition boosts CD8^+^ T cell growth and combines effectively with PD-1/PD-L1 inhibitors.^54^ In a biopsy-based study of NSCLC patients before and after immunotherapy, Gettinger et al. found that LAG-3 expression on tumor-infiltrating lymphocytes increased in 5 out of 8 tumors following treatment, and TIM-3 expression increased in 3 out of 8 cases. While other immunosuppressive molecules, such as TIGIT, were simultaneously upregulated, these changes did not reach statistical significance.^55^ These findings suggest that, in certain patients with acquired resistance to PD-1/PD-L1 blockade, compensatory upregulation of LAG-3 and, to a smaller extent, TIM-3 may occur during treatment.

**Figure 2:**
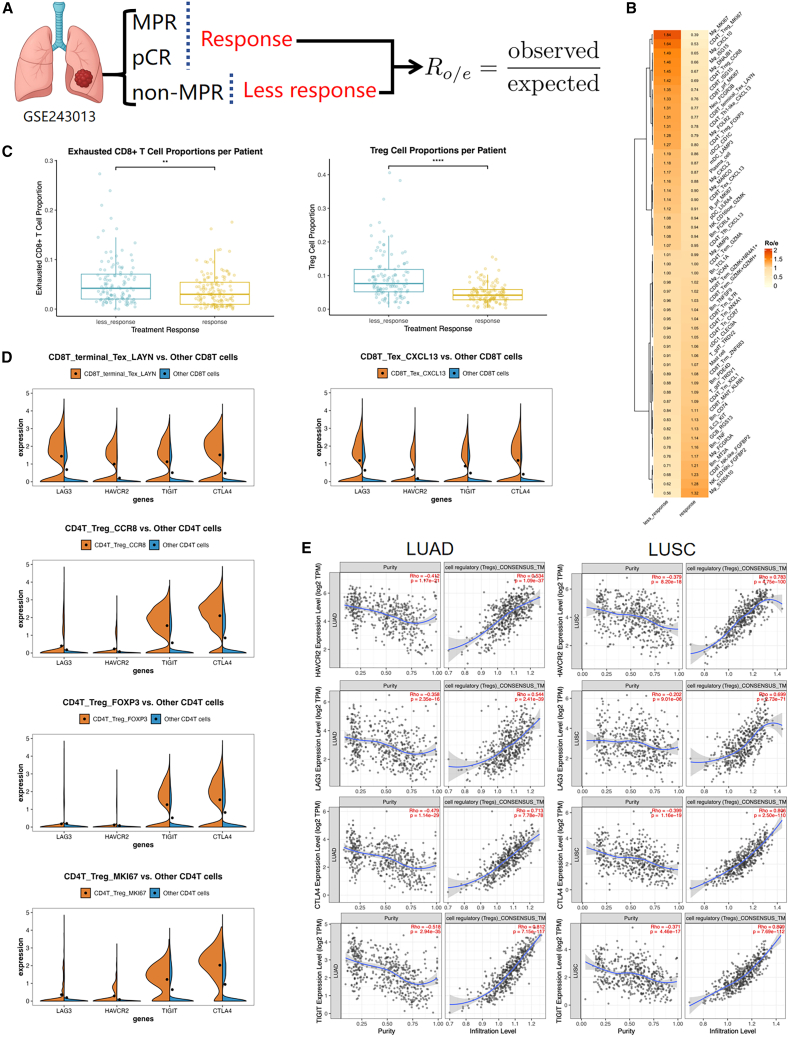
Upregulation of alternative immune checkpoints in NSCLC patients with poor response to PD-1/PD-L1 blockade (A) Study overview and enrichment analysis. Patients were classified into a response group, which contains pathological complete response (pCR, RVT = 0%) and major pathological response (MPR, RVT ≤10%), and a less-response group that contains non-MPR (RVT ≥10%). (B) Heatmap of *R*_*o/e*_ analysis. For each annotated cell type, *R*_*o/e*_ was computed within each group. (C) Comparison of exhausted CD8^+^ T cells and Tregs. Exhausted CD8^+^ T cells (CD8T_terminal_Tex_LAYN; CD8T_Tex_CXCL13) and Treg (CD4T_Treg_CCR8; CD4T_Treg_FOXP3; CD4T_Treg_MKI67) were aggregated per patient, ^∗∗^*p* < 0.01, ^∗∗∗∗^*p* < 0.0001. (D) Checkpoint-gene expression across T cell subtypes. Violin plots show the expression of LAG3, HAVCR2 (TIM-3), TIGIT, and CTLA4 between each index subset (orange) and the remaining CD8^+^ or CD4^+^ T cells (blue). (E) Correlation of checkpoint genes with Treg infiltration in bulk cohorts. TIMER3.0 “Purity-adjusted” analysis of LUAD and LUSC shows associations between HAVCR2 (TIM-3), LAG3, CTLA4, TIGIT expression, and Treg infiltration level (CONSENSUS_TME).

To validate these mechanisms in NSCLC, we re-analyzed the single-cell RNA-sequence dataset GSE243013 following the original methods (Figure 2).^56^^,^^57^ After quality control and random downsampling, patients were separated by pathological response (response: pCR or MPR, RVT ≤10%; less-response: non-MPR, RVT ≥10%). For each annotated cell cluster, we computed the ratio of observed to expected cell numbers (*R*_*o/e*_) within each group.^58^ Exhausted CD8^+^ T cells and multiple Treg subsets showed higher *R*_*o/e*_ in less-response. At the patient level, the aggregate proportion of the exhausted CD8^+^ T cells among CD45^+^ cells and Tregs among CD45^+^ cells was significantly increased in less-response. We then examine the checkpoint expression in five selected T cell subsets. In the exhausted CD8^+^ subsets, LAG3, HAVCR2 (TIM-3), TIGIT, and CTLA4 were clearly elevated. Across Treg subsets, CTLA4 and TIGIT showed marked enrichment, while LAG3 and HAVCR2 remained at low levels. Finally, in TCGA LUAD and LUSC bulk cohorts, these checkpoint expressions correlated positively with Treg infiltration in TIMER 3.0 under purity-adjusted analysis, supporting an association between checkpoint upregulation and an immunosuppressive TME.^59^ Detailed descriptions of the data processing and analytical procedures are provided in the data processing and analysis details section.

Collectively, resistance to PD-1/PD-L1 blockade in NSCLC arises from multifactorial and interrelated processes involving both tumor-intrinsic and microenvironmental components. Defective antigen presentation, T cell exclusion, infiltration of immunosuppressive cells, and compensatory upregulation of alternative immune checkpoints all contribute to impaired antitumor immunity and diminished responsiveness to PD-1/PD-L1 inhibitors. These mechanisms converge to shape an immunosuppressive “cold” TME, which undermines the efficacy of checkpoint blockade. Table 1 provides a concise overview of the major resistance mechanisms, the key molecular or cellular factors involved, and their immunological consequences as discussed above.

## CAR-T cells in NSCLC: From mechanistic framework to immunologic barriers

The advent of CAR T cell therapy has revolutionized cancer immunotherapy by enabling T cells to recognize and eliminate tumor cells independent of MHC restriction.^3^ While remarkable efficacy has been achieved in hematologic malignancies, translating CAR-T therapy into NSCLC has proven substantially more challenging.^6^ The biological complexity of NSCLC—including heterogeneous antigen expression, immunosuppressive TME, and T cell exhaustion mediated by inhibitory checkpoints such as PD-1—collectively hinders durable therapeutic responses.^6^ To contextualize the mechanistic and translational barriers underlying these challenges, this section provides a comprehensive overview of CAR-T cell structure and generational evolution, summarizes clinical and preclinical efforts targeting NSCLC-specific antigens, and examines how PD-1 signaling within the TME compromises CAR-T cell efficacy. Together, these aspects establish the mechanistic rationale for the subsequent discussion on combination strategies integrating CAR-T therapy with PD-1/PD-L1 blockade.

### Structural evolution and mechanistic basis of CAR-T cells

**Figure 3:**
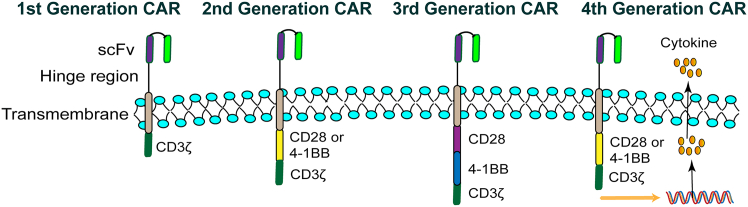
Structural evolution of CAR-T cells across four generations First-generation CARs consist of an scFv, a hinge region, a transmembrane domain, and a CD3ζ signaling module. Second-generation CARs add a single costimulatory domain such as CD28 or 4-1BB to enhance T cell activation and persistence. Third-generation CARs incorporate two costimulatory domains, enabling stronger and more coordinated signaling. Fourth-generation CARs include an inducible cytokine or immunomodulatory gene cassette activated through NFAT, allowing the engineered T cells to secrete effector molecules and modulate the TME.

The structural evolution of CAR-T cells represents a paradigm of iterative bioengineering to enhance anti-tumor efficacy (Figure 3). The first-generation CARs, featuring a solitary CD3ζ signaling domain, demonstrated limited clinical success due to inadequate T cell activation and persistence. A transformative advance came with second-generation constructs, which incorporated a single co-stimulatory domain (e.g., CD28 for potent activation or 4-1BB for enhanced persistence) alongside CD3ζ, providing the critical secondary signal required for robust T cell expansion, cytokine production, and long-term survival; these form the backbone of all currently approved CAR-T therapies. Subsequent efforts to amplify signaling further led to third-generation CARs harboring two distinct co-stimulatory domains, although their clinical superiority over second-generation designs remains equivocal.^60^ The field has since progressed to fourth-generation CAR-T cells (T cells redirected for antigen-unrestricted cytokine-initiated killing, also called TRUCKs), which are built upon second-generation backbones and engineered to inducibly express immunomodulatory agents (e.g., interleukin-12 (IL-12)) upon antigen engagement, thereby remodeling the immunosuppressive TME and recruiting innate immunity to tackle antigenically heterogeneous solid tumors.^61^

The development of CAR-T cell therapy has advanced into a phase of highly modular fifth-generation designs, aimed at holistically enhancing T cell potency, persistence, and safety. These next-generation constructs build upon the foundational architectures of earlier iterations—specifically, the integration of a single costimulatory domain (e.g., CD28 or 4-1BB) in second-generation CARs and the inducible immunomodulatory capacity (e.g., cytokine secretion) of fourth-generation TRUCKs.^60^ By incorporating novel membrane receptors and synthetic molecular modules, these designs employ distinct mechanistic pathways to overcome key clinical barriers. Representative strategies include the engineering of inverted cytokine receptors to convert suppressive signals in the TME into activating signals, thereby enhancing CAR-T cell fitness, and the development of bispecific CARs to mitigate tumor heterogeneity and antigen escape.^62^^,^^63^ Consequently, the field is now focused on the rational development of logic-gated systems, safety switches, and allogeneic “off-the-shelf” CAR-T cells, representing a concerted effort to address the persistent challenges of on-target/off-tumor toxicity, the immunosuppressive TME, and the logistical complexities of autologous cell therapies.^64^^,^^65^

### Current landscape of CAR-T therapeutic targets and clinical development in NSCLC

CAR-T cells target cancer antigens via MHC-independent mechanisms, generating ample tumor-reactive T cells, bypassing immune evasion from MHC downregulation.^66^ However, CAR-T cell therapy still faces significant limitations in the treatment of NSCLC. First, NSCLC lacks tumor antigens that exhibit the high specificity and widespread expression comparable to CD19, which is commonly found in hematological malignancies. Second, prolonged antigen exposure leads to the upregulation of inhibitory receptors on CAR-T cells, ultimately resulting in functional exhaustion. Finally, the potential development of cytokine release syndrome (CRS) and neurotoxicity further restricts the clinical application of CAR-T cell therapy in NSCLC.^67^^,^^68^ Nevertheless, several early-phase clinical trials are ongoing to evaluate the feasibility of CAR-T cells in NSCLC (Table 2).

**Table 2:** Clinical trials in treatment of NSCLC by CAR-T cells

Target antigen	Patient population	Sponsor	Phase	NCT number	Study start
CD44v9	Moderate or far advanced NSCLC	Beijing Immunochina Medical Science & Technology Co., Ltd.	Phase 1/Phase 2	NCT05117138	2022
CEA	Recurrent/advanced NSCLC with CEA^+^ and HLA-A∗02 LOH (Tmod-gated)	A2 Biotherapeutics Inc	Phase 1/Phase 2	NCT05736731	2023
	Advanced/metastatic/recurrent NSCLC	Chongqing Precision Biotech Co., Ltd	Phase 1	NCT06043466	2023
CLDN6	Metastatic or unresectable NSCLC with ≥50% tumor cells expressing ≥2+ CLDN6	BioNTech Cell & Gene Therapies GmbH	Phase 1	NCT04503278	2020
EGFR	Chemotherapy refractory EGFR^+^ NSCLC	Chinese PLA General Hospital	Phase 1/Phase 2	NCT01869166	2013
	Advanced EGFR^+^ NSCLC	Sun Yat-sen University	Phase 1	NCT04153799	2019
		Second Affiliated Hospital of Guangzhou Medical University	Early Phase 1	NCT05060796	2019
	Recurrent/advanced NSCLC with EGFR^+^ and HLA-A∗02 LOH (Tmod-gated)	A2 Biotherapeutics Inc	Phase 1/Phase 2	NCT06682793	2025
EphA2	Stage IV, EphA2^+^ NSCLC	Second Affiliated Hospital, School of Medicine, Zhejiang University	Phase 1	NCT06972576	2025
GD2	Stage IV NSCLC that is platinum-refractory and has received prior PD-1 and/or PD-L1 therapy	UNC Lineberger Comprehensive Cancer Center	Early Phase 1	NCT05620342	2023
GPC3	Advanced NSCLC with GPC3^+^ (≥50%, IHC)	Wondercel Biotech (ShenZhen)	Early Phase 1	NCT06653023	2024
MOv19-BBz	Metastatic/recurrent adenocarcinoma NSCLC with malignant pleural effusion	University of Pennsylvania	Phase 1	NCT07116057	2025
MSLN	Recurrent/advanced NSCLC with MSLN^+^ and HLA-A∗02 LOH (Tmod-gated)	A2 Biotherapeutics Inc	Phase 1/Phase 2	NCT06051695	2024
MUC1	Advanced MUC1^+^ NSCLC	PersonGen BioTherapeutics (Suzhou) Co., Ltd.	Phase 1/Phase 2	NCT02587689	2015
MUC1-C	Advanced/metastatic NSCLC of epithelial origin	Poseida Therapeutics, Inc.	Phase 1	NCT05239143	2022
TnMUC1	Advanced TnMUC1^+^ NSCLC	Kite, A Gilead Company	Phase 1	NCT04025216	2019
ROR1	Relapsed/refractory ROR1^+^ NSCLC	Lyell Immunopharma, Inc	Phase 1	NCT05274451	2022
	Metastatic or inoperable NSCLC with ROR1 expression >20%	Fred Hutchinson Cancer Center	Phase 1	NCT02706392	2016

CD44v9, CD44 variant containing exon v9; NSCLC, Non-small cell lung cancer; CEA, Carcinoembryonic antigen; CLDN6, Claudin-6; EGFR, Epidermal growth factor receptor; HLA-A∗02 LOH, HLA-A∗02 allele loss of heterozygosity; EphA2, Ephrin type-A receptor 2; GD2, Disialoganglioside 2; PD-1, Programmed cell death protein 1; PD-L1, Programmed death-ligand 1; GPC3, Glypican-3; IHC, Immunohistochemistry; MSLN, Mesothelin; MUC1, Mucin 1; MUC1-C, Mucin 1 - C-terminal subunit; TnMUC1, Tn-antigen form of mucin 1; ROR1, Receptor tyrosine kinase-like orphan receptor 1.

In NSCLC, the primary targets for CAR-T therapy include polymorphic epithelial mucin 1 (MUC1), EGFR, receptor tyrosine kinase like orphan receptor 1 (ROR1), carcinoembryonic antigen (CEA), and mesothelin (MSLN).^69^ A phase 1 study evaluated regional (intrapleural) administration of MSLN-targeted autologous CAR-T cells in patients with malignant pleural disease, including malignant pleural mesothelioma as well as metastatic lung and breast cancers with pleural involvement. Across the entire cohort, intrapleural CAR-T cell administration was feasible and generally well tolerated. In this combined population, disease stabilization lasting at least six months was observed in eight patients, and two patients experienced complete metabolic responses on PET imaging.^70^

EGFR mutations are common oncogenic drivers in NSCLC. In advanced EGFR-mutant NSCLC, more than 50% of cases exhibit PD-L1 expression, yet these tumors respond poorly to anti-PD-1/PD-L1 therapies.^71^ A phase 1 clinical trial explored the application of EGFR-targeted CAR-T cells in patients with advanced NSCLC. Post-treatment, detectable levels of EGFR-CAR-T cells were observed in the peripheral blood of 8 out of 9 participants with EGFR mutations. Notably, one patient exhibited a partial response lasting over 13 months, while six others achieved stable disease. The median overall survival was reported at 15.6 months.^72^

### TME and PD-1 signaling: Core obstacles to CAR-T cell efficacy

The immunosuppressive TME remains a formidable barrier to the success of CAR-T cell therapy in solid malignancies. A critical component of this suppression is mediated by the PD-1/PD-L1 axis: tumor and stromal cells often upregulate PD-L1, engaging PD-1 on CAR-T cells and delivering inhibitory signals that blunt their effector functions. Mechanistically, persistent PD-1 signaling restricts metabolic fitness by inhibiting glycolysis and mitochondrial respiration, triggering early dysfunction and driving exhaustion phenotypes.^73^ Moreover, recent studies have shown that the sensitivity of CAR-T cells to PD-1/PD-L1-mediated inhibition is strongly influenced by the affinity of their antigen-binding domain (single-chain fragment variables, scFv) and the choice of co-stimulatory domain. Andreu-Saumell et al. demonstrated that CAR-T cells with low-affinity antigen recognition are far more susceptible to suppression by PD-L1 than high-affinity counterparts. Genetic ablation of PD-1 by CRISPR significantly restored cytokine secretion and polyfunctionality in low-affinity but had little effect in high-affinity CAR-T cells; furthermore, co-stimulatory domains such as CD28 and ICOS accentuated this sensitivity, while 4-1BB-based CARs exhibited intrinsic resistance.^74^

In addition to direct signaling, PD-1-mediated metabolic reprogramming contributes to CAR-T cell decline. Studies report that PD-1 engagement downregulates PGC-1α, a master regulator of mitochondrial biogenesis, impairing mitochondrial integrity and long-term persistence.^75^ To counteract these deleterious effects, innovative “armoring” approaches have been developed. For instance, CAR-T cells engineered to secrete IL-10 resist exhaustion, preserve stem-like memory features, and mediate durable tumor clearance in solid tumor and metastasis models.^76^ Meanwhile, bioengineering strategies targeting metabolism further underscore the link between PD-1 signaling, mitochondrial dysfunction, and functional decline: Akhtar et al. found that CAR-T cell exhaustion, as indicated by PD-1 induction, correlates with a drop in mitochondrial respiratory capacity, suggesting metabolic support could restore potency.^77^ A particularly promising strategy integrates PD-1 blockade with local immunomodulation: Murad et al. engineered CAR-T cells to secrete a bifunctional αPD-L1-IL-12 fusion protein, which simultaneously blocks inhibitory checkpoints and delivers pro-inflammatory IL-12 in the tumor milieu. This design substantially enhances CAR-T trafficking, IFN-γ production, and antitumor efficacy *in vivo*, while minimizing systemic cytokine toxicity.^78^

In sum, PD-1 signaling not only imposes a brake on CAR-T cell activation but also reprograms their metabolism and drives exhaustion. Affinity tuning, co-stimulatory domain engineering, metabolic reprogramming, and fusion-protein “self-delivering” checkpoint blockade represent a multi-pronged toolkit to overcome these barriers. Continued translation of these strategies holds promise to improve the safety, persistence, and effectiveness of CAR-T therapies in solid tumors.

## Clinical application strategies of CAR-T therapy combined with PD-1/PD-L1 blockade in NSCLC

In 2013, John et al. first demonstrated the synergistic potential of combining CAR-T therapy with PD-1/PD-L1 inhibitors, showing that PD-1 blockade enhances the antitumor efficacy of anti-HER2 T cells.^79^ Clinical trials examining CAR-T cell therapy in combination with PD-1/PD-L1 axis blockade for the treatment of NSCLC have shown promise, with multiple studies currently underway (Table 3).

**Table 3:** List of Registered Clinical trials of CAR-T cell therapy combined with PD-1/PD-L1 axis inhibition for the treatment of NSCLC

NCT number	Mode of action	Target antigen	Clinical trial phase
NCT03182816	Secrete PD-1 antibodies	EGFR	Phase 1/2
NCT03179007	Secrete PD-1 antibodies	MUC1	Phase 1/2
NCT04489862	Secrete PD-1 antibodies	MSLN	Early Phase 1
NCT04556669	Secrete PD-1 antibodies	CD22	Phase 1
NCT02862028	Secrete PD-1 antibodies	HER3	Phase 1/2
NCT03198052	Secrete PD-1 antibodies	TGFβ	Phase 1
NCT03060343	Targeting PD-L1	PD-L1、CD80/CD86	Early Phase 1
NCT03525782	PD-1 knockout	MUC1	Phase 1/2
NCT03198052	PD-1 knockout	NKG2D	Phase 1

PD-1, Programmed cell death protein 1; EGFR, Epidermal growth factor receptor; MUC1, Mucin 1; MSLN, Mesothelin; CD22, Cluster of differentiation 22; HER3, Human epidermal growth factor receptor 3; TGFβ, Transforming growth factor-beta; PD-L1, Programmed death-ligand 1; CD80, Cluster of differentiation 80; CD86, Cluster of differentiation 86; NKG2D, Natural killer group 2, member D.

### Exogenous co-administration of CAR-T cells and PD-1/PD-L1 inhibitors

**Figure 4:**
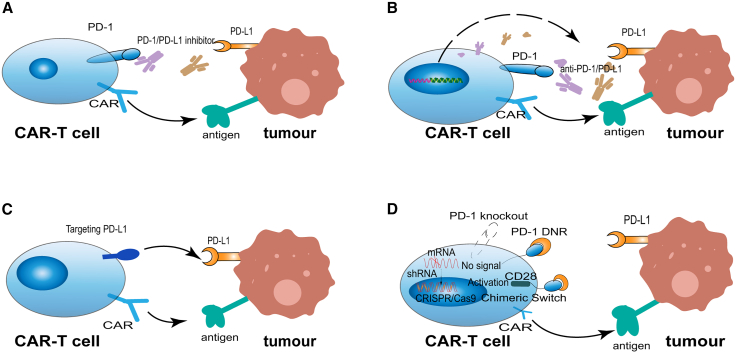
CAR-T cell-based strategies to enhance PD-1/PD-L1 blockade in NSCLC (A) Exogenous combination therapy: Co-administration of CAR-T cells with PD-1/PD-L1 inhibitors augments CAR-T activation, cytokine secretion, and antitumor cytotoxicity. This approach enhances CAR-T persistence and tumor clearance but may cause systemic immune-related toxicities and requires repeated dosing. (B) Autonomous antibody-secreting CAR-T cells: CAR-T cells engineered to locally secrete anti-PD-1 or anti-PD-L1 antibodies continuously block the PD-1/PD-L1 pathway within the TME. This strategy enables sustained checkpoint inhibition and reduces systemic exposure. (C) PD-L1-targeted CAR-T therapy: CAR-T cells constructed with PD-L1-specific scFv recognize and eliminate PD-L1^+^ tumor and immunosuppressive cells. This approach directly targets immune-evasive mechanisms in NSCLC and helps overcome tumor antigen heterogeneity. (D) Genetic disruption of PD-1 signaling: CAR-T cells engineered by PD-1 knockout (CRISPR-Cas9), DNR, or “PD1CD28” chimeric switch receptor effectively prevent inhibitory signaling and maintain effector function by converting PD-L1 binding into activating or neutral cues.

As numerous studies have demonstrated, the efficacy of CAR-T cell therapy is enhanced by combination with anti-PD-1/PD-L1 monoclonal antibodies. This combination appears to fine-tune activation-induced cell death, eliminate tumor cells, enhance survival rates, and prolong the persistence of adoptive cell therapies (Figure 4A).^8^^,^^70^^,^^79^ Research suggests that high levels of PD-L1 expression may impair the cytotoxic capacity of CAR-T cells against lung cancer, thereby compromising their anti-tumor efficacy. Blockade of the PD-1/PD-L1 axis with agents, such as pembrolizumab or cemiplimab potentiates cytokine release and augments CAR-T-mediated cytotoxicity. Animal studies demonstrate that the combination of pembrolizumab with CAR-T therapy effectively suppressed tumor growth in mouse models. Importantly, the administered treatment doses showed no notable effects on the animals’ body weight or signs of cytokine-related toxicity.^80^

Clinical data indicate that this combination therapy increases the objective response rate in patients with advanced disease, underscoring its therapeutic feasibility.^66^^,^^74^ Integrating CAR-T cell therapy with PD-1/PD-L1 checkpoint inhibitors provides a more targeted and adaptable dosing strategy while obviating the need for additional T cell genetic engineering. However, this approach has several limitations. Firstly, PD-1 blockade induces only a transient immunosuppressive effect, necessitating frequent administration to maintain efficacy. Furthermore, PD-1 inhibitors are at risk of sequestration by TAMs before reaching CAR-T cells, thereby attenuating their capacity to disrupt the PD-1/PD-L1 interaction. Perhaps most concerningly, systemic delivery of these inhibitors can trigger immune-related adverse events, which represents a major hurdle for this otherwise promising combination treatment.^81^ Consequently, the administration of anti-PD-1 therapy following CAR-T cell treatment does not consistently yield the anticipated synergistic effect. Interestingly, research has shown that CAR-T cells expanded with interleukin-7 (IL-7) and interleukin-15 (IL-15) exhibit a superior response to PD-1 blockade compared to those cultured with IL-2 and IL-7.^82^ Therefore, combining anti-PD-1 antibodies with CAR-T cells preconditioned with specific cytokine combinations could significantly improve treatment outcomes, potentially allowing for reduced antibody doses and mitigating adverse effects.

### CAR-T cells with PD-1/PD-L1 antibody-secreting capacity

Due to the suboptimal efficacy associated with the exogenous co-administration of CAR-T cells and PD-1/PD-L1 inhibitors in NSCLC, researchers are actively exploring alternative strategies. One innovative approach involves engineering CAR-T cells to directly secrete PD-1/PD-L1 inhibitors (Figure 4B).^83^ This method enables continuous, localized secretion of PD-1-blocking agents at the tumor site, thereby achieving persistent disruption of immune checkpoint signaling. A key advantage of this strategy is the extension of T cell functional persistence while circumventing the need for systemic administration of checkpoint inhibitors.^3^ This approach obviates the need for repeated systemic dosing by engineering CAR-T cells to locally express anti-PD-1/PD-L1 antibodies during therapy, thus preventing the adverse effects associated with systemic PD-1/PD-L1 blockade.

Building on the promising approach of dual-targeting immune checkpoints and the TGF-β pathway, scientists have engineered innovative CAR-T cells that produce bifunctional trap proteins directed against both PD-1 and TGF-β. *In vitro* and *in vivo* studies demonstrated that these enhanced CAR-T cells surpass conventional counterparts by more effectively suppressing immunosuppressive pathways, promoting T cell longevity and proliferation, and exhibiting superior anti-tumor efficacy without premature exhaustion. Notably, in murine tumor models, these modified CAR-T cells significantly enhanced tumor control and therapeutic outcomes, suggesting their potential as a transformative strategy for eradicating refractory solid tumors and preventing cancer recurrence.^84^

scFvs are the most widely used recognition domains in CAR constructs and can be engineered to enable CAR-T cells to secrete antibodies targeting tumor cell surface receptors (Figure 4B). Anti-PD-L1 scFv is one of the most commonly used engineered elements with CAR structures.^8^ By utilizing a dual-promoter lentiviral vector, human primary T cells can be induced to express both an anti-carbonic anhydrase Ⅸ CAR and anti-PD-L1 scFv, providing patients with a self-replicating and constitutively active biotherapeutic that continuously delivers PD-L1 scFv within the TME. This approach facilitates local accumulation of anti-PD-L1 scFv, resulting in sustained blockade of PD-1 signaling and maximizing the antitumor immune response, while minimizing off-target autoimmunity.^3^ The advantages of this strategy include prioritizing recruitment and targeting of tumor regions with high PD-L1 expression and protection of CAR-T cells from immune exhaustion, thereby optimizing their therapeutic efficacy. Despite these advancements, engineering CAR-T cells with scFVs is subject to constraints: scFv-integrated CARs may induce CAR clustering, potentially leading to CAR-T cell exhaustion.^85^^,^^86^

### CAR-T therapy targeting PD-L1

The efficacy of CAR-T therapy in NSCLC has been limited, primarily because of two key challenges: the absence of reliable tumor-specific antigen (TSA) and the highly immunosuppressive TME. Within this hostile milieu, cancer cells and immune-suppressive cells evade immune surveillance by overexpressing PD-L1, thereby directly inhibiting the activity of cytotoxic T cells. This mechanism has established PD-L1 as a prominent therapeutic target in NSCLC (Figure 4C). Lou et al. engineered PD-L1-targeted CAR-T cells using humanized anti-PD-L1 antibodies to simultaneously attack tumor and immunosuppressive cells. The therapy showed antigen-specific cytotoxicity in NSCLC models, eliminating both cancer and immunosuppressive cells.^87^ CAR-T cells targeting PD-L1 demonstrate a remarkable ability to eliminate NSCLC cells and reduce xenograft tumors, particularly in cases exhibiting elevated PD-L1 expression. Furthermore, administering a small, localized dose of radiation to NSCLC cells and tumors with low PD-L1 expression significantly enhances the efficacy of PD-L1-targeted CAR-T cells.^8^

CARPD-L1z, a CAR-encoding vector with a high-affinity scFv targeting human PD-L1, incorporates co-stimulatory (4-1BB and TLR2) and CD3ζ signaling domains. CARPD-L1z-engineered T cells effectively lyse PD-L1^+^ tumor cells *in vitro*, enhance cytokine production, and suppress NSCLC, gastric cancer, and hepatocellular carcinoma progression, but combined administration with CARMSLNz T cells shows no synergistic tumor inhibition.^88^ This result occurs because PD-L1 is expressed by both tumor cells and activated T cells.^89^ When tumor cells activate CARMSLNz T cells, they trigger an increase in PD-L1 expression, rendering these cells vulnerable to targeting by CARPD-L1z T cells in the xenograft model. Interestingly, CARPD-L1z T cells show no signs of self-reactivity. This can be explained by the close proximity of the anti-PD-L1 scFv and PD-L1 on the same cell membrane; this interaction induces PD-L1 internalization, thereby reducing its surface expression on CARPD-L1z T cells.^88^

### PD-1 disrupted CAR-T therapy

The PD-1/PD-L1 pathway is pivotal in tumor immune regulation. Tumor cells use it to deplete T cells. PD-1 inhibitors halt this pathway to avoid T cell exhaustion.^90^ Compared with other methods, genetically modifying CAR-T cells to block the PD-1/PD-L1 signaling pathway is a more accurate and effective method.

#### PD-1 knockout CAR-T therapy

Knocking out the PD-1 gene in CAR-T cells can significantly enhance T cell immune activity, thereby improving the clinical efficacy of CAR-T therapy in antitumor treatment (Figure 4D).^19^ Engineered CAR-T cells with PD-1 gene modifications maintain robust cytotoxicity and display significantly diminished exhaustion and dysfunction traits.^91^ In a clinical trial (NCT03525782), 20 patients with stage Ⅲb to Ⅳ NSCLC received at least one cycle of PD-1 knockout engineered anti-MUC1 CAR-T therapy. No grade 3 or higher toxicities of CRS were observed in the trial. Clinical evaluation revealed that 11 patients achieved stable disease, while 9 patients experienced disease progression. All patients reported significant symptom improvements after infusion. The number of circulating CAR-T cells gradually decreased after treatment, declining to 20% of initial levels after four months, indicating that repeated administration may be required to sustain therapeutic efficacy. These findings confirm the favorable safety and tolerability of PD-1 knockout CAR-T cells in NSCLC patients, although definitive evidence of clinical efficacy has not been established.

Knocking out PD-1 is one of the promising strategies to block the PD-1/PD-L1 pathway in CAR-T therapy. Some studies suggest that completely eliminating PD-1 may hinder CAR-T cell expansion and development, whereas temporarily blocking PD-1 expression enables strong *in vitro* proliferation while preserving anticancer effects. Researchers speculate that PD-1 could be crucial for regulating standard T cell growth and specialization—meaning its complete removal might inadvertently weaken T cell multiplication and reduce tumor-fighting potential.^92^ Kalinin et al. found that, compared to PD-1 silencing, PD-1 knockout leads to more rapid terminal differentiation and accelerated exhaustion of CAR-T cells, with a considerable decrease in their ability to multiply.^93^ The PD-1/PD-L1 signaling axis plays a pivotal role in modulating CAR-T cell function, influencing exhaustion, expansion, and programmed cell death. While some researchers advocate for PD-1 inhibition during CAR-T cell production, the optimal approach—whether through gene silencing or knockout—remains hotly debated and warrants deeper exploration.

#### CAR-T therapy using chimeric switch receptors

The “PD1CD28” chimeric switch receptor is constructed by fusing the extracellular domain of PD-1 to the transmembrane and intracellular domains of CD28 (Figure 4D).^94^ When the PD-1 component of this receptor binds to its ligand PD-L1, it delivers a stimulatory signal via the CD28 cytoplasmic domain, instead of transmitting an inhibitory signal through the PD-1 cytoplasmic domain. Although the “PD1CD28” chimeric switch receptor demonstrates potential in prolonging the persistence of CAR-T cells within the challenging environment of solid tumors, it does not independently activate T cells. It remains reliant on TSA recognition receptors to perform the essential functions required for effective therapeutic action.^88^ MSLC-directed CAR-T cells, modified with this chimeric switch receptor, markedly outperformed standard CAR-T cells or solo anti-PD-1 treatment in hindering tumor progression. Furthermore, the authors confirmed that while PD-1 blockade could increase CAR-T antitumor efficacy, CAR-T cells expressing the “PD1CD28” switch receptor demonstrated superior tumor control.^94^

In cancer patients, PD-1-positive T cells found in peripheral blood show heightened expression of genes linked to cytotoxicity and are heavily involved in T cell activation pathways, demonstrating potent tumor-killing effects against both the patient’s own cancer cells and established tumor lines. Despite their cytotoxic potential, these cells face therapeutic limitations due to their immunosuppressive nature and poor ability to proliferate. To overcome these hurdles, Zhang and colleagues engineered PD-1^+^ T cells by integrating a “PD1CD28” switch receptor along with a CD19 CAR, then expanded them *in vitro*. The resulting modified T cells exhibited robust proliferation and enhanced tumor-targeting capabilities in lab tests. In a clinical trial, four cancer patients were treated with infusions of these autologous PD-1/CD28-CD19 CAR T cells. The therapy was well tolerated, with no serious side effects reported. One patient experienced a complete remission lasting 6.7 months, while the other three maintained stable disease progression.^95^

#### PD-1 dominant negative receptor CAR-T therapy

PD-1 dominant negative receptor (DNR) CAR-T therapy represents a novel approach designed to enhance the therapeutic efficacy of CAR-T cell treatment in NSCLC by counteracting tumor-induced immune suppression. The underlying mechanism involves the incorporation of DNR into the CAR structure. This modified DNR retains only the extracellular domain of PD-1, which is responsible for PD-L1 binding, while lacking the intracellular signaling domain. As a result, it can competitively bind to PD-L1 expressed on tumor cells without triggering inhibitory signals, thereby effectively blocking the activation of the endogenous PD-1 pathway.^88^^,^^96^ This pertains to the engineering modification of membrane surface receptors, where its efficacy is determined by the number of DNRs expressed on the surface of CAR-T cells. It represents a “localized blockade” rather than complete ablation. Thus, its impact on CAR-T cell expansion is relatively insignificant.^92^

Qin et al.’s novel PD-1 DNR CAR-T design combines PD-1’s extracellular and transmembrane segments with a 4-1BB/TLR2 co-stimulatory domain and CD3ζ activation module, forming a fully functional chimeric antigen receptor. This structural design endows the CAR-T cells with dual functional advantages: it enables the induction of cytotoxic responses via the CD3ζ signaling domain, allowing for direct elimination of PD-L1^+^ tumors such as NSCLC; furthermore, by targeting PD-L1, a prevalent immune escape marker within the TME of solid tumors, it can overcome the issue of TSA loss caused by tumor heterogeneity, achieving tumor eradication independently of TSA-CAR signaling.^88^

Together, these strategies represent innovative attempts to integrate CAR-T therapy with PD-1/PD-L1 inhibition, each with distinct mechanistic advantages. Co-administration builds on clinically validated agents, antibody-secreting CAR-T cells provide localized immunomodulation, PD-L1-targeting CAR-T cells directly attack checkpoint ligand-expressing tumors, and PD-1-disrupted CAR-T cells maintain robust effector function. At the same time, these strategies still present some limitations, and addressing them requires tailored optimization approaches to enhance both safety and efficacy.

## Challenges and future directions of CAR-T therapy combined with PD-1/PD-L1 blockade

Exogenous co-administration of CAR-T cells with PD-1/PD-L1 inhibitors is conceptually straightforward and clinically accessible. Yet, systemic immune-related adverse events remain difficult to control. Optimization directions include localized drug delivery (e.g., nanoparticles, intratumoral injection) and personalized dosing regimens to reduce peripheral immune overactivation. CAR-T cells engineered to secrete PD-1/PD-L1 blocking agents provide a strategy to localize checkpoint inhibition to the tumor site. While this approach may minimize systemic exposure, efficacy depends on tightly regulated secretion. Excess release risks local toxicity, whereas insufficient release limits therapeutic benefit. Advanced systems, such as inducible promoters (e.g., nuclear factor of activated T cells (NFAT)-responsive elements) or SynNotch-based control circuits, may refine spatiotemporal regulation.^97^

For PD-L1 targeted CAR-T therapy, the advantage lies in its dual capacity to directly eliminate PD-L1^+^ tumor cells while also reversing local immunosuppression. However, the physiological expression of PD-L1 in normal tissues raises the risk of “on-target, off-tumor” toxicity. To address this, logic-gated CAR designs have been developed, in which T cell activation requires the co-expression of PD-L1 together with another tumor-specific antigen, thereby improving selectivity.^98^ In the case of PD-1-disrupted CAR-T cells, studies indicate that either knocking out PD-1 or introducing PD-1/CD28 switch receptors can markedly enhance CAR-T persistence and function. Yet, sustained and complete blockade of PD-1 may disturb immune homeostasis. Consequently, current optimization strategies emphasize partially reversible inhibition and inducible switch-receptor expression, aiming to balance long-term efficacy with reduced risk of autoimmunity.

### Precision targeting: Overcoming tumor heterogeneity via dual/multi-targeting and personalized antigen profiling

Antigen heterogeneity remains a central barrier to effective CAR-T therapy in NSCLC, as tumor cells can escape immune pressure through dynamic modulation, downregulation, or selective loss of target antigens. Dual-targeting CAR designs have emerged as a critical strategy to address this limitation. Bispecific or tandem CARs frequently function through an OR-gate mechanism, in which recognition of either antigen is sufficient to activate T cells, thereby preserving cytotoxicity even when one antigen becomes partially or completely downregulated under therapeutic pressure.^6^ In contrast, AND-gate architectures—commonly implemented via split CARs that require simultaneous engagement of two antigens—offer enhanced tumor specificity and reduces off-tumor activation by restricting full T cell activation to cells co-expressing both targets. Together, these complementary dual-target strategies provide a more resilient framework against antigen escape, strengthen antitumor potency through cooperative signaling, and broaden therapeutic applicability across tumors with heterogeneous or unstable antigen profiles.^99^

To elucidate the translational potential of this strategy for NSCLC, three specific dual-targeting paradigms warrant particular attention. First, the combination of EGFR and Met addresses a critical resistance mechanism in lung cancer. Since MET amplification frequently serves as a bypass track for EGFR-mutant tumors, simultaneous blockade is essential.^100^ This logic has been clinically validated by the phase 3 MARIPOSA study (NCT04487080), where the EGFR/MET bispecific antibody amivantamab demonstrated superior efficacy over monotherapy. This success provides a robust biological rationale for developing EGFR/c-Met bispecific CAR-T cells to intercept immune escape. Second, strategies targeting HER2 and MUC1 illustrate the utility of complementary signaling. In these tandem CAR designs, HER2 engagement drives potent cytotoxicity while MUC1 signaling supports metabolic fitness and persistence, thereby ensuring the elimination of tumor variants with variable antigen density.^101^ Finally, recent clinical data targeting EGFR and IL13Rα2 have provided proof-of-concept that bivalent CAR-T cells can effectively manage high spatial heterogeneity in solid tumors, a principle directly applicable to the complex antigen landscape of NSCLC.^102^ Together, these examples demonstrate that Boolean “OR” gating is not merely a theoretical construct but a clinically viable approach to broaden the therapeutic window.

Additionally, in NSCLC cases with concomitant dysregulated signaling pathways, the development of CARs designed to target these aberrant pathways represents a compelling frontier in therapy. Based on the hyperactivation of the Wnt signaling pathway and the high expression of frizzled-7 (FZD7) in NSCLC, we developed a targeted antibody (SHH002-hu1)^103^; it has demonstrated potent antitumor efficacy in preclinical models, both alone and in combination with a PD-L1 inhibitor. Additionally, we have developed a bispecific protein, SHH002-hu1-MICA, that targets FZD7. Our research demonstrated that this agent leverages the MICA-NKG2D axis to effectively redirect NK cells, thereby mediating the potent killing of triple-negative breast cancer cells.^104^ These findings collectively indicate that bispecific designs using FZD7 as an anchor point represent an effective strategy to counteract tumor immune escape, thereby providing a solid theoretical basis that informs the development of our “FZD7-X” dual-targeting CAR-T cells.

### Reinforcing T cell fitness: Reprogramming the immunosuppressive and metabolic TME

Even when CAR-T cells successfully infiltrate tumor sites, their function can still be progressively impaired by the immunosuppressive TME and harsh metabolic constraints, ultimately leading to exhaustion and diminished antitumor efficacy. To overcome this barrier, several layers of engineering strategies have been explored. First, “armored” CAR-T designs can locally reverse immunosuppression. CAR-T cells engineered with NFAT-responsive promoters to secrete PD-1/PD-L1 scFv or other immune activators upon activation can establish a localized immunostimulatory milieu.^97^^,^^105^ For example, GPC2-targeted CAR-T cells armed with NFAT-inducible, membrane-tethered IL-15/interleukin-21 (IL-21) significantly enhanced antitumor activity against neuroblastoma without systemic cytokine toxicity.^106^ Such a design enhances both CAR-T cells and endogenous tumor-infiltrating T cells via autocrine and paracrine effects, while minimizing systemic toxicities associated with widespread immune checkpoint blockade.

Beyond immunomodulation, recent efforts have focused on metabolic and epigenetic reprogramming to endow CAR-T cells with enhanced resistance to exhaustion and sustained functionality. On the metabolic side, a recent study demonstrated that enforced expression of engineered PGC-1α in CAR-T cells promotes mitochondrial fitness and memory-like properties, thereby significantly improving their persistence and antitumor activity in solid tumor models.^107^ At the epigenetic level, Lynn et al. reported that overexpression of the transcription factor c-Jun in CAR-T cells reshapes gene regulatory networks and counteracts exhaustion-associated transcriptional programs, delaying terminal differentiation and preserving memory-like features, which ultimately enhances CAR-T cell persistence within the TME.^108^ These findings suggest that reinforcing intrinsic T cell fitness represents a key direction for sustaining durable responses.

### Safeguarding CAR-T therapy: Addressing clinical safety concerns and mitigation strategies

Although combination therapies can markedly enhance antitumor efficacy, they also increase the risk of systemic toxicities, most notably CRS and immune effector cell-associated neurotoxicity syndrome (ICANS), as well as “on-target, off-tumor” damage. Therefore, addressing these safety concerns is paramount for clinical translation, necessitating a dual focus on pharmacological management and engineering safety switches into cellular products.^65^^,^^109^

CRS and ICANS represent the primary acute complications, driven by a systemic surge of inflammatory cytokines (e.g., IL-6, IFN-γ).^110^ Clinically, these acute toxicities are primarily managed through pharmacological interventions, including the anti-IL-6 receptor antibody Tocilizumab and corticosteroids, which rapidly dampen the systemic inflammatory response.^109^^,^^110^ In parallel, embedding controllable safety switches into cellular products holds significant clinical value for providing an immediate “emergency brake” against severe acute toxicities.^65^ One well-studied approach involves integrating an inducible apoptotic switch (e.g., inducible caspase 9) into CAR constructs, whereby administration of a small-molecule dimerizer (such as AP1903/rimiducid) enables the rapid elimination of most switch-bearing T cells, thus providing an immediate “emergency brake” against severe acute toxicities.^111^

The risk of “on-target, off-tumor” damage, which results from CAR-T cell recognition of the target antigen expressed at low levels on vital healthy tissues, necessitates engineering strategies that enhance tumor-specific recognition.^109^ Beyond complete elimination via suicide genes, new CAR designs have been developed to introduce logic-gating to improve specificity.^112^ The full activation of these safety-enhanced CARs often depends on integrated multiple signals. This can be achieved through AND-gate architectures, which require simultaneous antigen recognition, or through inhibitory CARs that engage “safety” antigens on healthy tissue to deliver a potent inhibitory signal.^65^

On the other hand, localized delivery represents another key strategy for improving safety by physically restricting CAR activity to the disease site. Previous studies have shown that lipid nanoparticles can deliver CAR mRNA directly into T cells *in vivo*, thereby inducing their conversion into functional CAR-T cells and achieving effective target clearance.^113^ Similarly, small extracellular vesicles carrying CAR mRNA have been demonstrated to activate local immunity or generate CAR effector cells *in situ* within lung cancer models, thereby strengthening the tumor immune microenvironment.^114^ These advances suggest that lung-targeted delivery of CAR mRNA via lipid nanoparticles or small extracellular vesicles may induce the *in situ* generation of CAR effector cells or enhance local immune activation, thereby reducing systemic exposure and minimizing off-target toxicity.^115^ Given the transient nature of mRNA expression, this approach adds an additional layer of controllability. Together, pharmacological management, advanced CAR engineering (including safety switches and logic-gating), and localized delivery platforms represent indispensable tools for translating combined CAR-T and PD-1/PD-L1 therapies into the clinic.

## Conclusions

PD-1/PD-L1 inhibitors, which operate by shutting down immune checkpoint signals to revive T cell functionality, have achieved significant advances in the management of NSCLC. CAR-T therapy, with its MHC-independent recognition and potent cytotoxicity, offers a complementary approach. The integration of CAR-T with PD-1/PD-L1 blockade represents a promising strategy to overcome resistance mechanisms and improve clinical outcomes.

Emerging approaches, including exogenous co-administration, antibody-secreting CAR-T cells, PD-L1-targeting CAR-T constructs, and PD-1-disrupted CAR-T cells, highlight the versatility of this therapeutic paradigm. Nonetheless, significant challenges persist, such as antigen heterogeneity, T cell exhaustion, systemic toxicities, and safety concerns. It is foreseeable that future optimization will not remain limited to isolated advances within single technical dimensions, but will instead progress toward the integrated application of multi-pronged and multi-layered strategies. In this context, comprehensive solutions are required, encompassing multi-target CAR designs, reprogramming of the TME, incorporation of safety switches, and localized delivery systems.

In summary, the integration of CAR-T therapy with PD-1/PD-L1 blockade presents a promising new paradigm for overcoming resistance in NSCLC. Its successful translation will rely on multi-pronged strategies: overcoming tumor heterogeneity via multi-targeting CARs, reinforcing T cell fitness through “armoring” and metabolic reprogramming of the immunosuppressive TME, and, crucially, mitigating safety concerns such as CRS, neurotoxicity, and on-target/off-tumor effects by incorporating safety switches, logic-gated CAR designs, and localized delivery platforms. The effective management of these safety challenges will be pivotal for the broad clinical adoption of this combination therapy.

In this process, artificial intelligence technology is expected to play a crucial enabling role. For example, our current study has already applied machine learning methods to uncover potential associations between immune checkpoint upregulation and the immunosuppressive state of the TME. These findings demonstrate artificial intelligence’s practical value in biomarker discovery, mechanistic analysis, and the development of therapeutic strategies.

Looking ahead, machine learning can be further leveraged to screen potential targets, predict synergistic effects of multi-target approaches, and optimize localized delivery strategies. Such applications have the potential to enhance the precision, safety, and controllability of combination therapies, providing a powerful tool for both mechanistic exploration and clinical translation.

With the continuous accumulation of clinical trial evidence and ongoing refinement of key technologies, the combination of CAR-T therapy and PD-1/PD-L1 blockade is poised to gradually establish a central role in the NSCLC treatment landscape. This progress is likely to propel oncology immunotherapy toward a more efficient, intelligent, and personalized paradigm, ultimately delivering more durable and safer clinical benefits for patients.

## Acknowledgments

This review was supported by the 10.13039/100007219Natural Science Foundation of Shanghai (23ZR1427400); “AI Empowerment for Research Program” initiated by the 10.13039/501100003395Shanghai Municipal Education Commission (SHJWAIJK241205); Key clinical program of 10.13039/100017950Shanghai Municipal Health Commission (20214Y0516); key supportive specialized construction project for the Health and Wellness System in Jinshan District, Shanghai (JSFCZK202102).

## Author contributions

X.L. conceived and wrote the manuscript and served as the primary contributor. Z.W. contributed equally as co-first author, playing a key role in translation, data processing, and manuscript revision. S.M. was responsible for structural optimization and organization of the manuscript framework. Y.Z. and J.Y. participated in literature collection and data compilation. J.P., X.X., and J.P. conducted comprehensive bibliographic research and supported data acquisition. B.L. provided administrative and organizational support throughout the project. All authors reviewed and approved the final version of the manuscript.

## Declaration of interests

The authors declare no competing interests.

## Declaration of generative AI and AI-assisted technologies in the writing process

During the preparation of this work, the authors used ChatGPT in order to improve language. After using this tool, the authors reviewed and edited the content as needed and take full responsibility for the content of the publication.
